# Prognostic and predictive value of examined lymph node count in stage III colorectal cancer: a population based study

**DOI:** 10.1186/s12957-024-03404-7

**Published:** 2024-06-13

**Authors:** Ran Wei, Zifan Zheng, Qinghai Li, Yan Qian, Chong Wu, Yin Li, Mian Wang, Jianhui Chen, Weiling He

**Affiliations:** 1 Gastrointestinal Surgery Center, The First Affiliated Hospital, Sun Yat-sen University, Guangzhou, Guangdong 510080 China; 2 Department of Vascular Surgery, The First Affiliated Hospital, Sun Yat-sen University, Guangzhou, Guangdong 510080 China; 3 Department of General Surgery, Guangxi Hospital Division of The First Affiliated Hospital, Sun Yat-sen University, Nanning, China; 4 Department of Gastrointestinal Surgery, Xiang’an Hospital of Xiamen University, School of Medicine, Xiamen University, Xiamen, Fujian 361000 China

**Keywords:** Examined lymph node, Colorectal cancer, Tumor microenvironment, Restricted cubic spline curves, Prognosis

## Abstract

**Background:**

The role of tumor-draining lymph nodes in the progression of malignant tumors, including stage III colorectal cancer (CRC), is critical. However, the prognostic and predictive value of the number of examined lymph nodes (ELNs) are not fully understood.

**Methods:**

This population-based study retrospectively analyzed data from 106,843 patients with stage III CRC who underwent surgical treatment and registered in three databases from 2004 to 2021. The Surveillance, Epidemiology, and End Results (SEER) cohort was divided using into training and test cohorts at a ratio of 3:2. We employed restricted cubic spline (RCS) curves to explore nonlinear relationships between overall survival (OS) and ELNs counts and performed Cox regression to evaluate hazard ratios across different ELNs count subtypes. Additional validation cohorts were utilized from the First Affiliated Hospital, Sun Yat-sen University and The Cancer Genome Atlas (TCGA) under the same criteria. Outcomes measured included OS, cancer-specific survival (CSS), and progression-free survival (PFS). Molecular analyses involved differential gene expression using the “limma” package and immune profiling through CIBERSORT. Tissue microarray slides and multiplex immunofluorescence (MIF) were used to assess protein expression and immune cell infiltration.

**Results:**

Patients with higher ELNs counts (≥ 17) demonstrated significantly better long-term survival outcomes across all cohorts. Enhanced OS, CSS, and PFS were notably evident in the LN-ELN group compared to those with fewer ELNs. Cox regression models underscored the prognostic value of higher ELNs counts across different patient subgroups by age, sex, tumor differentiation, and TNM stages. Subtype analysis based on ELNs count revealed a marked survival benefit in patients treated with adjuvant chemotherapy in the medium and large ELNs counts (≥ 12), whereas those with fewer ELNs showed negligible benefits. RNA sequencing and MIF indicated elevated immune activation in the LN-ELN group, characterized by increased CD3+, CD4+, and CD8 + T cells within the tumor microenvironment.

**Conclusions:**

The number of ELNs independently predicts survival and the immunological landscape at the tumor site in stage III CRC, underscoring its dual prognostic and predictive value.

**Supplementary Information:**

The online version contains supplementary material available at 10.1186/s12957-024-03404-7.

## Introduction

Colorectal cancer (CRC) is a major contributor to cancer-related morbidity and mortality worldwide [1]. Accurate staging of lymph nodes (LNs) is critical for determining the oncological outcomes and guiding the postoperative therapeutic strategies in CRC patients [2–4]. In particular, the presence of metastatic lesions in regional mesenteric LNs decisively necessitates the use of adjuvant chemotherapy (AC) [2–4]. Research has consistently shown that the number of examined lymph nodes (ELNs) is intimately linked to patient prognosis, with the American Joint Committee on Cancer (AJCC) recommending a minimum of 12 ELNs be assessed to optimize CRC staging [5].

The prognostic significance of ELNs extends beyond mere nodal involvement; a higher count of ELNs independently predicts improved survival outcomes in CRC patients [6–8]. This correlation is often attributed to the “stage migration” effect or the Will Rogers phenomenon, where increased LN yields can alter the pathological N classification by identifying metastatic LNs that might otherwise be missed, potentially leading to either an upward or downward shift in stage classification [9, 10]. Such detailed LN evaluation is crucial not only for accurate staging but also for informing clinical decisions [10, 11], particularly given the substantial risk of recurrence in AJCC stage II–III CRC, which can reach up to 30% [12, 13]. These recurrences are frequently linked to micro-metastatic clones present at the initial surgery and may manifest clinically after prolonged periods of remission [14]. There is a scientific consensus that dormant cancer cells can intermittently resume proliferation, which may lead to recurrences even after extended periods of remission. These cells, present in minute amounts, might remain in proximity to the removed LNs or even spread to distant organs. Therefore, it is imperative to determine the optimal number of ELNs for removal and pathological analysis is imperative to decrease the risk of leaving metastatic LNs behind [15, 16].

From an immunological standpoint, regional LNs are central to antitumor immunity as the primary sites of T-cell activation [17]. These nodes engage in several antitumor activities, including the recruitment of antigen-presenting cells, initiation of adaptive immune responses, and maintenance of immunological memory by harboring tumor antigens and central memory cells [18–23]. Recent studies have reinforced a molecular link between increased ELN counts and stronger immune responses, indicating that a robust antitumor immune environment significantly contributes to the observed survival benefits with higher ELN counts [24]. This finding highlights the potential shortcomings of traditional LN dissection techniques in leveraging the full prognostic potential of LN staging, due to their limited capacity in assessing immune activation.

Given the complexities associated with determining the optimal number of ELNs solely from single-center or small-scale studies, our study introduces a novel subclassification system based on ELNs counts within a large, population-based dataset. This comprehensive study is designed to examine the relationship between ELNs count and long-term prognosis, including cancer stage migration in stage III CRC patients. By conducting detailed clinicopathological and immunological profiling, we aim to improve our understanding of the prognostic and predictive significance associated with various ELNs count subtypes.

## Methods

### Study populations

Our study involved a comprehensive review and analysis of CRC patients who underwent curative surgery and were registered in the Surveillance, Epidemiology, and End Results (SEER) database from January 1, 2004, to January 1, 2021. Inclusion criteria for the database encompassed patients aged between 18 and 85 years, diagnosed with pathological stage III disease according to the 8th edition of the AJCC staging criteria, and who received R0 (complete) resection. Exclusion criteria included individuals with a secondary cancer diagnosis or those suffering from immune disorders or autoimmune diseases. From the SEER database, a total of 104,078 patients were identified and included in the study. The CRC patient cohort from the SEER database was subsequently divided into two groups: a training cohort, comprising 60% of the patients, and a test cohort, consisting of the remaining 40%, using the ‘caret’ package in R software [25] (Table 1).

**Table 1:** The baseline characteristics of stage III colorectal cancer patients across diverse cohorts

Characteristics	Train cohort	Test cohort	FAH-SYSU cohort	TCGA cohort
	(*n* = 62,447)	(*n* = 41,631)	(*n* = 2622)	(*n* = 143)
Age at diagnosis, *n* (%), years old				
20–49	7489 (11.99)	4955 (11.90)	574 (21.89)	25 (17.48)
50–69	26,982 (43.21)	18,082 (43.43)	1437 (54.81)	63 (44.06)
70+	27,976 (44.80)	18,594 (44.66)	611 (23.30)	55 (38.46)
Gender, *n* (%)				
Female	31,163 (49.90)	20,720 (49.77)	1505 (57.40)	77 (53.85)
Male	31,284 (50.10)	20,911 (50.23)	1117 (42.60)	66 (46.15)
Year of CRC diagnosis, *n* (%)				
2004–2008	19,378 (31.03)	13,026 (31.29)	555 (21.17)	62 (43.36)
2009–2013	18,419 (29.50)	12,232 (29.38)	375 (14.30)	81 (56.64)
2014–2021	24,650 (39.47)	16,373 (39.33)	1692 (64.53)	0 (0.00)
Tumor location, *n* (%)				
Right colon	31,906 (51.09)	21,336 (51.25)	598 (22.81)	63 (44.06)
Left colon	19,418 (31.10)	12,965 (31.14)	918 (35.01)	35 (24.48)
Rectum	11,123 (17.81)	7330 (17.61)	1106 (42.18)	45 (31.47)
Tumor grade, *n* (%)^a^				
Well/moderately	45,723 (73.22)	30,555 (73.39)	1911 (80.91)	-
Poorly/undifferentiated	16,724 (26.78)	11,076 (26.61)	451 (19.09)	-
Histology type, *n* (%)^b^				
Adenocarcinoma	48,673 (77.94)	32,628 (78.37)	2190 (92.68)	121 (84.62)
Others	13,774 (22.06)	9003 (21.63)	173 (7.32)	22 (15.38)
Tumor size, *n* (%), cm ^c^				
< 3	11,148 (17.85)	7447 (17.89)	320 (12.35)	-
<=3&<5	24,066 (38.54)	16,148 (38.79)	1135 (43.79)	-
>=5	27,233 (43.61)	18,036 (43.32)	1137 (43.87)	-
Primary tumor, *n* (%)				
Yes	52,331 (83.80)	34,866 (83.75)	2622 (100.00)	143 (100.00)
No	10,116 (16.20)	6765 (16.25)	0 (0.00)	0 (0.00)
Pathological T stage, *n* (%)				
T1/2	8321 (13.32)	5596 (13.44)	269 (10.26)	12 (8.39)
T3/4	54,126 (86.68)	36,035 (86.56)	2353 (89.74)	131 (91.61)
Pathological N stage, *n* (%)				
N1	40,554 (64.94)	26,914 (64.65)	1864 (71.09)	90 (62.94)
N2	21,893 (35.06)	14,717 (35.35)	758 (28.91)	53 (37.06)
Adjuvant radiotherapy, *n* (%)				
No	56,522 (90.51)	37,670 (90.49)	2622 (100.00)	114 (91.20)
Yes	5925 (9.49_	3961 (9.51)	0 (0.00)	11 (8.80)
Adjuvant chemotherapy, *n* (%) ^d^				
No/unknown	23,518 (37.66)	15,721 (37.76)	320 (16.30)	-
Yes	38,929 (62.34)	25,910 (62.24)	1643 (83.70)	-
Median ELNs count (IQR)	2 (13–24)	17 (13–24)	19 (13–26)	19 (14–27)
Median positive ELNs count (IQR)	2 (1–5)	2 (1–5)	3 (2–3)	3 (1–5)

*Note* Data are number of patients; data in parentheses are percentage unless otherwise indicated. ^a^260 patients lacking of clinical features, ^b^ 260 patients lacking of clinical features, ^c^ 30 patients lacking of clinical features and ^d^ 559 patients lacking of clinical features in FAH-SYSU cohort

*Abbreviations* CRC, colorectal cancer ELNs, examined lymph nodes

To establish validation cohorts, patients represented in The First Affiliated Hospital, Sun Yat-sen University (FAH-SYSU) database and The Cancer Genome Atlas (TCGA) were selected under the same inclusion and exclusion criteria presented above. The purpose of the FAH-SYSU database is to collect and maintain data on CRC patients who have been diagnosed and treated at FAH-SYSU. The primary data in FAH-SYSU database include follow-up information, treatment information, and other medical examination data in addition to basic patient data. We analyzed data for consecutive CRC patients who underwent radical surgery between January 1, 2004, and January 1, 2021, in the FAH-SYSU database; 2,622 stage III CRC patients in the FAH-SYSU database were ultimately included in this study (Table 1).

We collected publicly available gene expression data from the TCGA database, along with gene mutation data and related clinical annotations, on May 1, 2023. All data for the 143 stage III CRC patients included in this study were obtained from the TCGA-COAD and TCGA-READ datasets (Table 1). We downloaded the RNA sequencing data (fragments per kilobase transcript million mapped reads (FPKM) values) from the TCGA database. This study was approved by the Institutional Ethics Committee of The First Affiliated Hospital, Sun Yat-sen University (Ethical code: [2024]282).

### Clinical characteristics and oncological outcomes

The following clinical characteristics were recorded and analyzed in SEER, FAH-SYSU and TCGA database: age at diagnosis, year of CRC diagnosis, gender, tumor location, tumor size, tumor pathological type, tumor differentiation, pathological TNM stage, adjuvant therapy and number of harvested lymph nodes. Oncological outcomes that were compared included: overall survival (OS), cancer-specific survival (CSS) and progression free survival (PFS) among SEER, FAH-SYSU and TCGA database. OS was determined from the date of CRC diagnosis to the date of death from any cause. CSS refers to the likelihood of patients surviving a specific type or stage of cancer within a certain period after diagnosis or treatment, excluding death from the cancer itself. PFS was described as the duration from CRC diagnosis to the time of cancer progression or death from any cause.

### Differentially expressed genes

Differentially Expressed Genes (DEGs) were assessed by using the “limma” R package, which applies linear models to microarray data. Statistically significant genes in sequencing were obtained by adjusted p value < 0.05 and an absolute fold change > 1.5.

### Gene set enrichment analysis

We conducted gene set enrichment analysis using curated gene sets from the Gene Ontology (GO) and the Kyoto Encyclopedia of Genes and Genomes (KEGG), which were obtained from the MSigDB database. To evaluate the enrichment levels of gene sets in tumor tissues across different ELN groups, we employed the Mann-Whitney U test. A corrected P value of less than 0.05 was considered the threshold for determining statistical significance.

### Immune cells infiltration estimations

Immune cell infiltration was estimated with transcriptomic data using TIMER and CIBERSORT. The xCell method (https://xcell.ucsf.edu/) was used to evaluate infiltrating levels of 64 immune and stroma cell types. Twenty-eight immune cell gene sets were collected from previous publications, and the ssGSEA method was used to quantify 28 infiltrating immune cells. We used the T cell exhaustion (TEX) signature, immune checkpoint inhibitory (ICI) signature and cytotoxic signature to assess T cell dysfunction and exclusion level based on the ssGSEA method.

### Tissue microarray slides construction

To enable standardized and concurrent assessment of protein expression across multiple tissue specimens, we constructed tissue microarrays slides for subsequent immunohistochemical (IHC) staining. In brief, we procured tumor tissue samples, which were then fixed in ethanol and embedded in paraffin. A senior pathologist meticulously evaluated hematoxylin and eosin (H&E)-stained sections—specifically, one random block per patient—to delineate the tumor regions most representative of the pathology. Subsequently, from each designated region, we extracted three 1 mm diameter tissue cylinders. Utilizing a Manual Tissue Arrayer (Beecher Instruments, Silver Spring, MD, USA), we carefully punched out these cylinders from the chosen donor blocks and methodically arranged them into a recipient paraffin block. We then prepared serial sections with a thickness of 4 μm and placed them onto silane-coated slides, which were primed for IHC staining. The final IHC staining analysis was performed on tissue cores from a cohort of 80 CRC patients represented in the FAH-SYSU database.

### IHC staining and multiplex immunofluorescence (MIF)

Primary CRC specimens were collected during surgery and subjected to meticulous formalin fixation and paraffin embedding. The prepared tissue blocks were sectioned at a thickness of 4 μm. These sections were subjected to a series of preparative procedures, including deparaffinization, rehydration, and quenching of endogenous peroxidase activity. For antigen retrieval, sections were treated with sodium citrate buffer (10 mM, pH 6.0) and heated in a microwave at 95 °C for 20 min. IHC staining was performed using an automated immunostainer (BenchMark ULTRA, Ventana Medical Systems, Inc.). The antibodies-CD3 were purchased from Abcam (dilution 1:800, SP7, Cambridge, Britain), antibodies-CD4 were purchased from Abcam (dilution 1:500, EPR6855, Cambridge, Britain) and antibodies-CD8 were purchased from Abcam (dilution 1:300, EPR21769, Cambridge, Britain). All antibodies were used to stain human tonsil tissues as positive controls, and isotype-matched antibodies were used to stain the same tissues as negative controls. IHC staining was performed with an automated immunostainer (BenchMark ULTRA, Ventana Medical Systems, Inc.).

### Statistical analysis

The association between two continuous variables was evaluated through the Pearson correlation test. To determine the hazard ratio (HR) and 95% confidence interval (CI) for assessing risk, both univariate and multivariate Cox proportional hazards regression analyses were applied. Kaplan-Meier survival analysis, supplemented by the log-rank test, was utilized to contrast survival rates between two or more groups, employing the ‘survminer’ R package (version 0.4.9). Restricted cubic spline (RCS) regression with knots, derived from multivariate Cox models, was implemented to ELNs counts alongside dose-response and nonlinear relationships. Propensity score matching (PSM) analysis, maintaining a 1:1 ratio, was adopted to mitigate potential biases in baseline clinical features between groups receiving postoperative adjuvant chemotherapy (Yes-AC) and those not (No-AC), within ELNs-related subtypes. Differences in immune cell populations and immune signatures between two and among three groups were assessed using the Mann-Whitney U test and the Kruskal-Wallis test, respectively. For categorical data, the chi-square test or Fisher’s exact test was employed. Statistical analyses were conducted using R software (version 4.2.2), with all tests being two-tailed. A p-value of less than 0.05 was deemed statistically significant.

## Results

### Patient characteristics

In this study, 62,447 stage III CRC patients were assigned to train cohort, 41,631 patients were assigned to test cohort from SEER database, 2,622 patients from FAH-SYSU database and 143 from TCGA database were analyzed in this study. We compared baseline characteristics of CRC patients from these databases (Table 1). The cumulative 1-, 3, 5 years OS were 86.2%, 68.8% and 57.2% in train cohort, and were 86.1%, 68.7% and 57.1% in test cohort, were 95.5%, 77.9% and 67.4% in FAH-SYSU cohort and 90.9%, 73.5% and 50.2% in TCGA cohort.

### Prognostic impact of the ELNs count for stage III CRC patients

In conducting clinicopathological analyses, both univariate and multivariate Cox proportional hazards regression models were utilized to forecast OS across the examined cohorts (Supplementary Tables 1–3). The results suggested that tumor location, size, pathological type, differentiation status, pathological TNM stage, administration of adjuvant therapy, and ELNs count were positively associated with OS in stage III CRC patients in the training cohort, test cohort, FAH-SYSU cohort and TCGA cohort (*P* < .05 for all). Aligning with these observations across the overall cohort, an increased ELNs count was distinctly associated with improved OS outcomes within both the N1 and N2 subgroups (*P* < .05), as detailed in Supplementary Table 4.

### Innovative subtype designation for patients with III CRC based on the ELNs counts

**Fig. 1:**
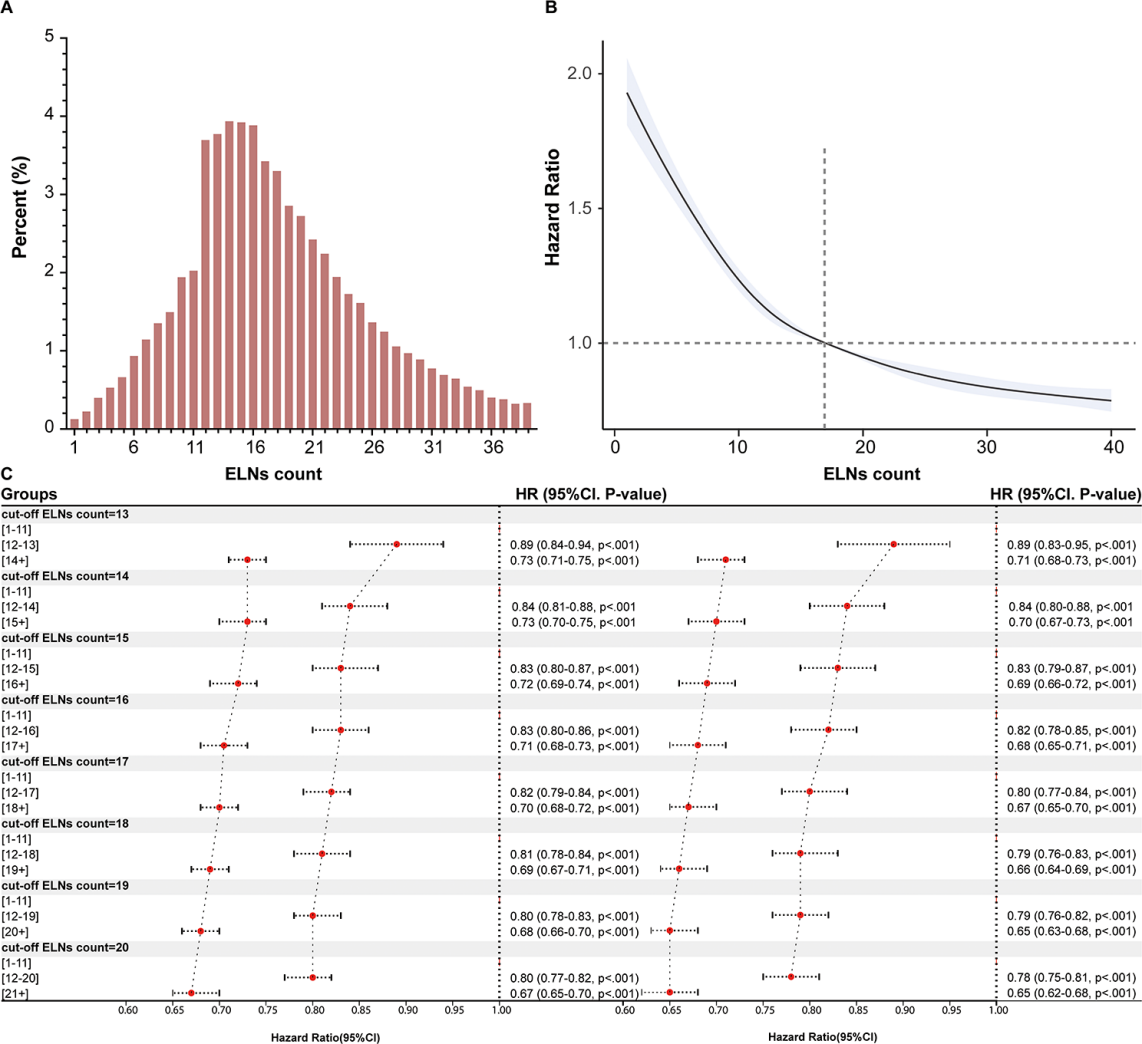
Innovative subtype for III CRC based on number of examined lymph nodes (ELNs). (**A**) Distribution of stage III CRC patient population across varyingELNs count categories. (**B**) A restricted cubic spline graph depicting the relationship between ELNs count and overall survival (OS). The graph illustrates the adjusted hazard ratio (HR, represented by the solid line) and the 95% confidence interval (CI, shaded in blue), demonstrating the association between the number of ELNs and OS in CRC patients. Lower HR correspond to improved survival outcomes. (**C**) derived from univariable Cox regression analysis for OS and cancer-specific survival (CSS) across distinct ELNs count thresholds, and the ELNs counts ≤ 12 serve as the reference group for comparison

For a more thorough evaluation, a Cox regression model with RCS was developed to identify an innovative subtyping strategy and show the link between OS risk and the ELNs count in patients with stage III CRC. The results of RCS analyses supported the established cut-off values for ELNs (Fig. 1A-B). The results of the RCS model adjusted for age at CRC diagnosis, sex, tumor location, tumor size, pathological type, differentiation status, and pathological T stage revealed a significant linear association between the ELNs count and OS in stage III CRC patients (P_overall_<0.001, P_nonlinear_<0.001) (Fig. 1B and Supplementary Fig. 1). The 50th quintile of the ELNs count was 16. Consistent with the results observed in the overall cohort, this ELNs cut-off was noted in the right colon, left colon and rectal cancer subgroups (Fig. 1C). The AJCC have established a standard yield of at least 12 ELNs for CRC. Figure 1B shows different cut-off values for the ELNs yield in predicting OS. To ensure survival and achieve accurate representation and generalizability, we suggest using 16 ELNs as the ideal yield cut-off, as determined by the RCS curve (Fig. 1C). The stage III CRC patients were then divided into three groups: the small number of ELNs (SN-ELN; ELNs count ≤ 11), medium number of ELNs (MN-ELN; 12 ≤ ELNs count < 16), and large number of ELNs (LN-ELN; ELNs count ≥ 17) groups. In both univariate and multivariate Cox regression analyses, the ELNs-related subtype was associated with OS in all cohorts (*P* < .05) (Table 2, Supplementary Tables 5–8).

**Table 2:** Multivariable Cox regression analysis in stage III colorectal cancer patients based on OS in different cohorts

Characteristics	Multivariable Cox in train cohort	Multivariable Cox in test cohort	Multivariable Cox in FAH-SYSU cohort	Multivariable Cox in TCGA cohort
Age at diagnosis (years old)				
20–49	Ref	Ref	Ref	Ref
50–69	1.38 (1.31–1.44, *p* < .001)	1.27 (1.20–1.35, *p* < .001)	1.21 (0.98–1.51, *p* = .082)	2.36 (0.50-11.06, *p* = .277)
70+	2.70 (2.57–2.83, *p* < .001)	2.50 (2.36–2.65, *p* < .001)	1.76 (1.36–2.28, *p* < .001)	4.67 (1.04–21.02, *p* = .045)
Gender				
Female	Ref	Ref	Ref	Ref
Male	1.18 (1.15–1.20, *p* < .001)	1.16 (1.12–1.19, *p* < .001)	0.88 (0.74–1.05, *p* = .155)	0.43 (0.21–0.90, *p* = .025)
Tumor location				
Right colon	Ref	Ref	Ref	Ref
Left colon	0.88 (0.85–0.90, *p* < .001)	0.89 (0.86–0.92, *p* < .001)	0.87 (0.67–1.14, *p* = .308)	
Rectum	1.00 (0.97–1.03, *p* = .954)	1.03 (0.98–1.07, *p* = .224)	1.48 (1.17–1.86, *p* = .001)	
Tumor grade				
Well/moderately	Ref	Ref	Ref	Ref
Poorly/undifferentiated	1.30 (1.27–1.33, *p* < .001)	1.28 (1.24–1.32, *p* < .001)	1.73 (1.38–2.18, *p* < .001)	
Histology type				
Adenocarcinoma	Ref	Ref	Ref	Ref
Others	1.08 (1.05–1.11, *p* < .001)	1.05 (1.02–1.09, *p* = .002)		
Tumor size (cm)				
< 3	Ref	Ref	Ref	Ref
<=3&<5	1.11 (1.07–1.15, *p* < .001)	1.13 (1.08–1.18, *p* < .001)	0.82 (0.61–1.09, *p* = .168)	
>=5	1.23 (1.19–1.28, *p* < .001)	1.26 (1.21–1.32, *p* < .001)	1.09 (0.81–1.46, *p* = .574)	
Pathological T stage				
T1/2	Ref	Ref	Ref	Ref
T3/4	1.65 (1.58–1.72, *p* < .001)	1.64 (1.56–1.73, *p* < .001)	1.87 (1.28–2.72, *p* = .001)	
Pathological N stage				
N1	Ref	Ref	Ref	Ref
N2	1.60 (1.56–1.64, *p* < .001)	1.58 (1.54–1.63, *p* < .001)	1.89 (1.57–2.27, *p* < .001)	3.23 (1.53–6.80, *p* = .002)
Adjuvant chemotherapy				
No/unknown	Ref	Ref	Ref	Ref
Yes	0.49 (0.47–0.50, *p* < .001)	0.49 (0.48–0.51, *p* < .001)	0.69 (0.56–0.86, *p* = .001)	
ELNs count				
ELNs related subtypes	Ref	Ref	Ref	Ref
MN-ELN	0.80 (0.77–0.82, *p* < .001)	0.79 (0.76–0.82, *p* < .001)	0.74 (0.58–0.94, *p* = .014)	0.58 (0.22–1.50, *p* = .260)
LN-ELN	0.67 (0.65–0.69, *p* < .001)	0.64 (0.61–0.66, *p* < .001)	0.65 (0.52–0.81, *p* < .001)	0.20 (0.08–0.55, *p* = .002)

*Note* Cox regression analyses are used to calculate the hazard ratio (HR) and 95% confidence interval (CI) based on OS. Covariates achieving statistical significance in univariable Cox regression analysis (*P* < .05) are subsequently incorporated into the multivariable analysis

*Abbreviations* CRC, colorectal cancer ELNs, examined lymph nodes; SN-ELN, small number of ELNs; MN-ELN, medium number of ELNs; LN-ELN, large number of ELNs

### Prognostic impact of the ELNs-related subtype in stage III CRC patients

**Fig. 2:**
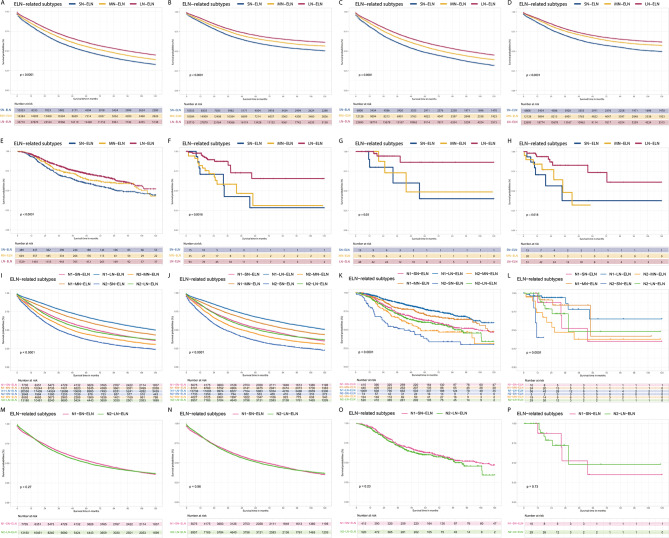
The prognostic impact of ELNs-related subtypes on stage III CRC patients. KM curves of the (**A**) a small number ELNs (SN-ELN) ≤ 11,12 ≤ a medium number of ELNs (MN-ELN) ≤ 16 and 17 ≤ a large number of ELNs (LN-ELN) for overall survival (OS) in train cohort; (**B**) cancer-specific survival (CSS) in train cohort; (**C**) OS in test cohort; (**D**) CSS in test cohort; (**E**) OS in FAH-SYSU cohort; (**F**) OS in TCGA cohort; (**G**) CSS in TCGA cohort; (**H**) progression free survival (PFS) in TCGA cohort. And then, the survival analysis was used to evaluated the OS for patients with both N1 stage and LN-ELN (N1-LN-ELN), N1-MN-ELN, N1-SN-ELN, both N2 stage and LN-ELN (N2-LN-ELN), N2-MN-ELN and N2-SN-ELN in (**I**) train cohort, (**J**) test cohort, (**K**) FAH-SYSU cohort and (**L**) TCGA cohort. The survival analysis was used to evaluate the OS for patients with N2-LN-ELN and N1-SN-ELN in (**M**) train cohort, (**N**) test cohort, (**O**) FAH-SYSU cohort and (**P**) TCGA cohort

Survival analyses for stage III CRC patients were conducted according to ELNs-related subtypes. Kaplan-Meier (K-M) curves indicated superior OS and CSS for the LN-ELN group compared to the MN-ELN and SN-ELN groups within the training cohort, with the following HRs and CIs: MN-ELN vs. SN-ELN: HR = 0.83, 95% CI = 0.80–0.86, *P* < .001; LN-ELN vs. SN-ELN: HR = 0.71, 95% CI = 0.68–0.72, *P* < .001 for OS (Fig. 2A) ; and MN-ELN vs. SN-ELN: HR = 0.82, 95% CI = 0.78–0.85, *P* < .001; LN-ELN vs. SN-ELN: HR = 0.68, 95% CI = 0.65–0.71, *P* < .001 for CSS (Fig. 2B). To corroborate the prognostic significance of ELNs-related subtyping, comparisons of OS, CSS, and progression-free survival (PFS) were extended to the LN, MN, and SN-ELN groups across the test, FAH-SYSU, and TCGA cohorts. These K-M analyses reaffirmed that patients in the LN-ELN group consistently exhibited better OS, CSS, and PFS than those in the MN-ELN and SN-ELN groups, with statistical significance observed across the board (MN-ELN vs. SN-ELN and LN-ELN vs. SN-ELN, *P* < .05), as detailed for OS (Fig. 2C) and CSS (Fig. 2D) in test cohort, OS (Fig. 2E) in FAH-SYSU cohort, OS (Fig. 2F), CSS (Fig. 2G) and PFS (Fig. 2H) in TCGA cohort.

To ascertain the significance of ELNs-related subtyping in patients with pathological N1 and N2 staging, we assessed the OS across the LN-, MN-, and LN-ELN groups within various cohorts. Patients with a higher ELNs count had better OS in both the N1 and N2 stage subgroups. Across the four cohorts, patients in the both N1 stage and LN-ELN (N1-LN-ELN) subgroup had the best OS, while those in the both N2 stage and LN-ELN (N2-LN-ELN) subgroup had the least favorable OS in train cohort (Fig. 2I), in test cohort (Fig. 2J), in FAH-SYSU cohort (Fig. 2K), and TCGA cohorts (Fig. 2L) (*P* < .05). Comparative analysis revealed no significant OS differences between patients in the N2-LN-ELN and N1-SN-ELN subgroups across the training (Fig. 2M), testing (Fig. 2N), FAH-SYSU (Fig. 2O) and in TCGA cohort (Fig. 2P) (*P* < .05), despite the association of pathological N stage with OS in both univariate and multivariate Cox regression analyses (Table 2). These results suggest that an elevated ELNs count might mitigate the adverse effects of LN metastasis on survival.

**Fig. 3:**
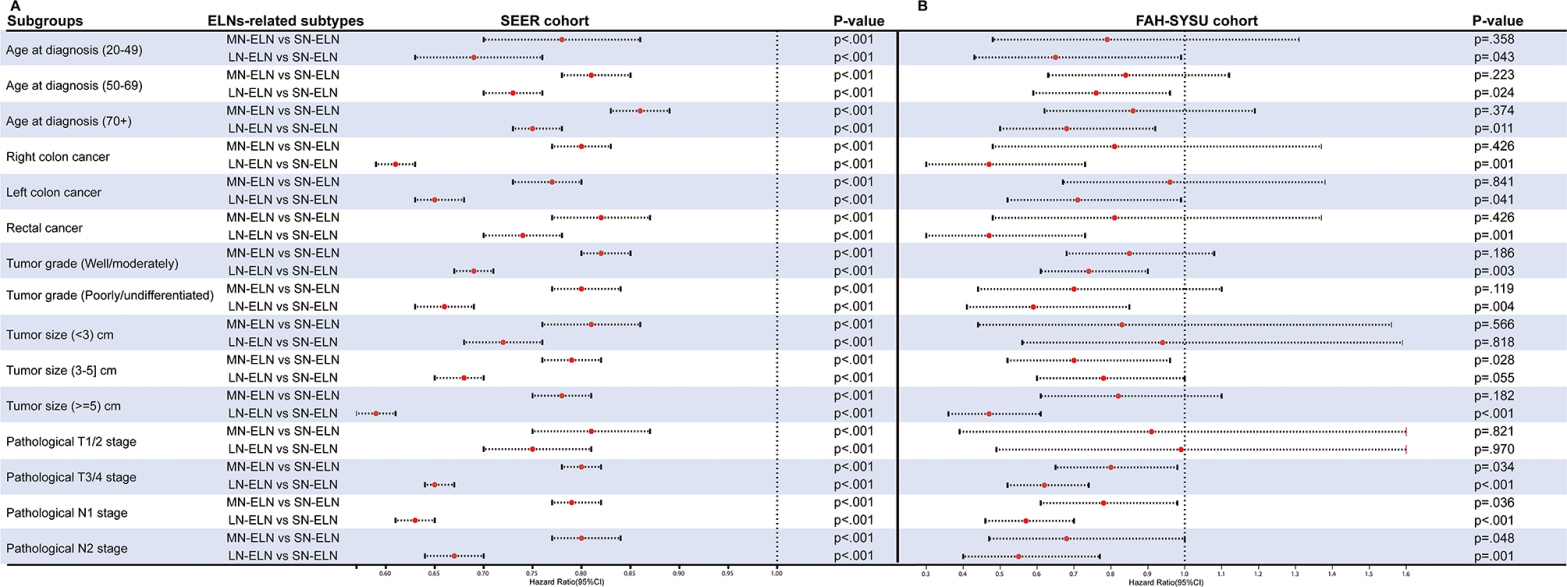
Subgroup analyses of ELNs-related subtypes of stage III CRC patients in SEER and FAH-SYSU cohort. Forest plot showing the factors associated with overall survival (OS) of stage III CRC patients with ELNs-related subtypes in (**A**) SEER and (**B**) FAH-SYSU cohort

Additionally, subgroup survival analyses were performed based on the three ELNs-related subtype groups. Consistent with the results observed in the overall cohort, the LN-ELN status was significantly related to better OS for different age, sex, tumor differentiation and TNM stage subgroups in the SEER and FAH-SYSU cohorts (*P* < .05) (Fig. 3A-B).

**Fig. 4:**
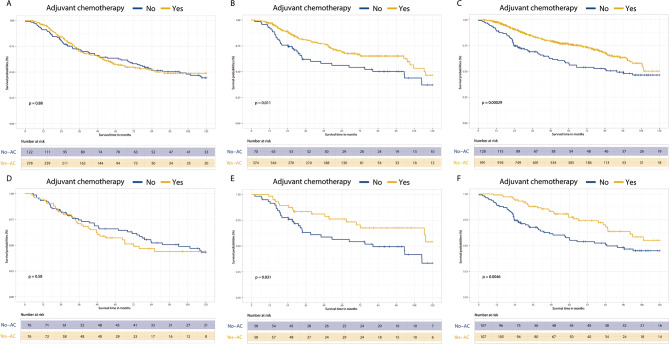
The survival analysis between receiving postoperative adjuvant chemotherapy (Yes-AC) and not receiving postoperative adjuvant chemotherapy (No-AC) group within different ELNs related subtypes group before and after propensity score matching (PSM) based on the FAH-SYSU cohort. To lessen selection bias, PSM matched the baseline parameters of the two groups in a ratio of 1:1, including age at diagnosis, gender, tumor location, tumor grade, histology type, tumor size, pathological T and N stage. (**A**) Overall survival (OS) comparison between YES-AC and No-AC group within SN-ELN before PSM. (**B**) OS comparison between YES-AC and No-AC group within MN-ELN before PSM. (**C**) OS comparison between YES-AC and No-AC group within LN-ELN before PSM. (**D**) OS comparison between YES-AC and No-AC group within SN-ELN after PSM. (**E**) OS comparison between YES-AC and No-AC group within MN-ELN after PSM. (**F**) OS comparison between YES-AC and No-AC group within LN-ELN after PSM

To delve deeper into the impact of the ELNs count on the efficacy of AC in stage III CRC patients, we examined the prognosis following AC treatment versus no AC treatment within the LN, MN and SN-ELN groups in the FAH-SYSU cohort (Fig. 4A-F). Comparisons of the baseline characteristics of stage III CRC patients in the FAH-SYSU cohort were performed between the Yes-AC group and the No-AC group before and after PSM (Supplementary Tables 9–11). Notably, in the LN-ELN group, a significant difference in OS was identified between the Yes-AC and No-AC groups both pre- and post-PSM (HR = 0.58, 95% CI = 0.43–0.78, *P* < .001 before PSM (Fig. 4C); HR = 0.52, 95% CI = 0.33–0.83, *P* = .005 after PSM (Fig. 4F)). A similar trend was observed in the MN-ELN group, where significant OS differences were noted between the Yes-AC and No-AC groups before and after PSM (HR = 0.60, 95% CI = 0.41–0.89, *P* = .012 before PSM (Fig. 4B); HR = 0.53, 95% CI = 0.29–0.95, *P* = .034 after PSM (Fig. 4E)). Conversely, within the SN-ELN group, no significant OS disparities were found between the Yes-AC and No-AC groups, neither before nor after PSM (HR = 0.98, 95% CI = 0.71–1.34, *P* = .879 before PSM (Fig. 4A); HR = 1.14, 95% CI = 0.72–1.80, *P* = .578 after PSM (Fig. 4D)), suggesting that AC may not enhance prognosis for stage III CRC patients with a low ELNs count.

### Evaluation of tumor microenvironment (TME) characteristics among the three ELNs-related subtypes

**Fig. 5:**
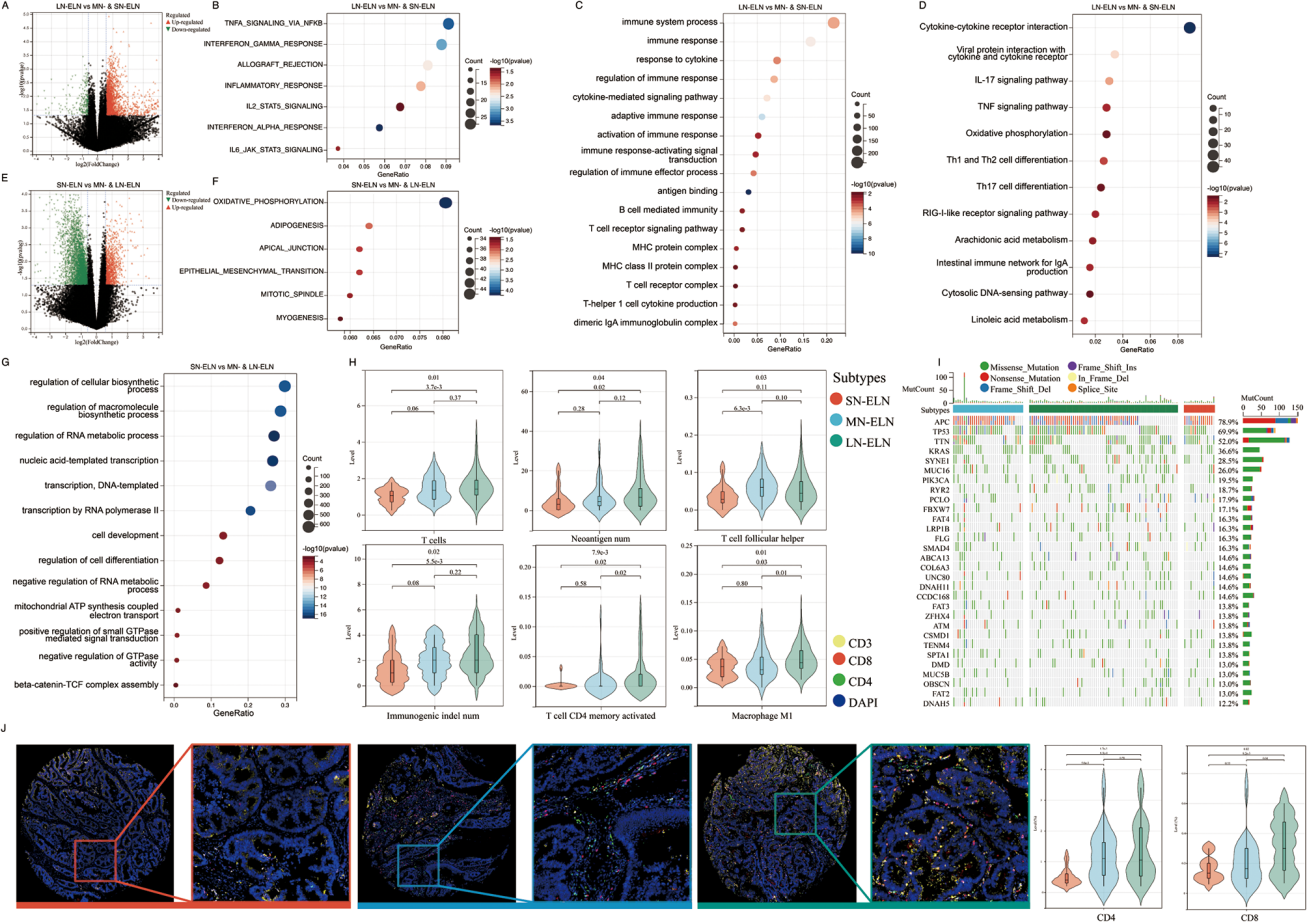
The analysis on potential biological mechanisms among different ELNs-related subtypes. (**A**) The volcano plot showed the differentially expressed genes (DEGs) between LN-ELN and MN- and SN-ELN groups in TCGA cohort. Red points: up-expression DEGs in LN-ELN with log2-fold change > 0.5 and *P* < .05; Green points: down-expression DEGs in LN-ELN with log2-fold change < -0.5 and *P* < .05; Black point: gene expression with |log2-fold change| < 0.5 or *P* < .05. The (**B**) Hallmarker, (**C**) GO and (**D**) KEGG analyzed presented the enrichment biological pathways between LN-ELN and MN- and SN-ELN groups. (**E**) The volcano plot showed the DEGs between SN-ELN and MN- and LN-ELN groups. The (**F**) Hallmarker and (**G**) GO analyzed presented the enrichment biological pathways between SN-ELN and MN- and LN-ELN groups. (**H**) The level of tumor microenvironment cell infiltration among LN-, MN- and SN-ELN groups by using the RNA-seq sequencing in TCGA cohort. (**I**) The characters of gene mutation among LN-, MN- and SN-ELN groups by using the DNA-seq sequencing in TCGA cohort. (**J**) The level of CD4 + T cell and CD8 + T cell compared among LN-, MN- and SN-ELN groups by using multiplex immunofluorescence method in FAH-SYSU cohort. The level of CD4 + T cell and CD8 + T cell on serial tissue microarrays

To discern the transcriptomic variations among stage III CRC patients categorized into the LN-, MN- and SN-ELN groups, RNA sequencing data from tumor samples within the TCGA cohort were analyzed. This analysis entailed comparing gene expression in tumor tissues between the LN-ELN group and the combined MN- and SN-ELN groups, as well as between the SN-ELN group and the combined MN- and LN-ELN groups. The comparison indicated that in the LN-ELN group, there were 2243 genes identified as upregulated and 228 as downregulated (Fig. 5A). Advanced comparisons to elucidate the biological distinctions between the LN-ELN group and the others (MN-ELN and SN-ELN) involved Gene Ontology (GO), hallmark gene set, and Kyoto Encyclopedia of Genes and Genomes (KEGG) enrichment analyses. These analyses highlighted significantly upregulated genes within the LN-ELN group (Fig. 5B-D), showcasing enriched terms and pathways related to immune response-activating signal transduction, B-cell-mediated immunity, T-cell receptor signaling pathway, and differentiation of Th1 and Th2 cells. Conversely, the comparison involving the SN-ELN group against the MN- and LN-ELN groups revealed 989 upregulated and 3100 downregulated genes in the SN-ELN group (Fig. 5E), with GO, hallmark gene set, and KEGG enrichment analyses pointing to enrichment in terms related to cell development and differentiation within the SN-ELN group (Fig. 5F-G).

To investigate the variances in immune cell composition among the SN-, MN- and LN-ELN groups, we employed a deconvolution approach using CIBERSORT and Gene Set Variation Analysis (GSVA) tools (Fig. 5H). The results indicated pronounced differences in TME. Specifically, the quantities of T cells, follicular helper T cells, activated memory CD4 + T cells, M1 macrophages, and the neoantigen load were notably higher in the LN-ELN group compared to the SN- and MN-ELN groups (Fig. 5H). Additionally, the gene mutations were observed within these three groups (Fig. 5I).

To refine our understanding of immune cell infiltration in stage III CRC patients across the ELNs-related subtypes, MIF was used to quantify CD3+, CD4 + and CD8 + T lymphocytes within tumor tissues (Fig. 5J). The analysis indicated notable increases in the numbers of CD3+, CD4 + and CD8 + T cells in the LN-ELN subgroup compared with the SN-ELN subgroup.

## Discussion

Tumor-draining LNs are secondary lymphoid organs that serve as immunological foci to regulate host adaptive immune responses [2–5]. However, the traditional clinical understanding that tumor cells invade regional LNs is based on the common view of the lymphatic system as a simple and passive route for sequential tumor cell metastasis and deposition [26–28]. Recently, a gradual mechanistic understanding of the crosstalk between immune and tumor cells has been gained, revealing that regional LNs, as a dynamic and complex compartment of the regional TME, could provide important insight into the relationship between tumor metastasis and host antitumor immunity [29]. Therefore, in contrast to the current clinical paradigm for tumor invasion, regional LNs should be considered a rich source of potential immunological biomarkers that reflect the state of host antitumor immunity regarding the prevention of regional and systemic tumor spread in clinical practice. However, current clinical settings often overlook the potential role of lymph nodes in evaluating antitumor immunity, and no method is yet available to evaluate antitumor immunological activation based on the features of LNs.

This study utilized three cohorts with the objectives of developing an ELNs-related subtype classification to assess the antitumor immune status and to predict survival outcomes using objective LN parameters for stage III CRC patients. Our approach introduces a pioneering model for segregating CRC patients based on immunological considerations tied to LN characteristics. Additionally, it was observed that the presence of CD3 + and CD8 + T cells within primary CRC sites positively correlates with the ELNs count. This association is biologically plausible, reinforcing the mechanistic underpinnings that lend scientific credibility to our model.

Recently, increasing evidence has shown that regional LNs, as reservoirs of activated tumor-specific immune cells, are the key regulators and indicators of the antitumor immune response and control the efficacy of immunotherapy [30, 31]. Although T cells can be activated and expanded in the TME, T cells are initially primed at regional LNs and then home to the tumor site through the lymphatic and blood circulation [32, 33]. Existing research has indicated that the elimination of regional LN or the obstruction of lymphocyte migration from these nodes does not halt tumor progression, underscoring the critical role of regional LNs in fostering antitumor immunity [34]. In alignment with these insights, our analysis demonstrated that the TME in patients within the LN-ELN category showed a heightened presence of immune cells, notably CD3 + and CD8 + T cells. This observation suggests a robust molecular linkage to an intensified antitumor immune response. Such a vigorous antitumor response is correlated with enhanced patient survival outcomes, corroborating findings from prior research [35–37]. Collectively, these insights offer a foundational mechanistic understanding of our model and compellingly elucidate the favorable prognosis observed in patients classified within the LN-ELN subgroup.

Nodal staging is crucial for prognosis estimation and therapy recommendation in CRC patients. In addition to the detection of metastases in LNs, the number of ELNs itself has prognostic implications, especially in patients with stage III CRC [38–40]. Despite the limitations of the current understanding of the prognostic impact of the LN count, a potential association between the immune response and the number of LNs has been recognized [41–43]. Similarly, previous research has demonstrated that the quantity of harvested leukemic neutrophils is favorably correlated with lymphocytic and inflammatory cell infiltration in the TME, which improves survival [44, 45]. The effect of the local antitumor immune response could lead to LN enlargement [46]. Therefore, these findings suggest that the number of ELNs could be used a surrogate marker of the antitumor immune response.

In the present study, we developed a clinically useful ELNs-related subtype classification to evaluate the antitumor immune response and to predict the prognosis of stage III CRC patients. However, this investigation is subject to several limitations. Initially, it is a retrospective cohort study focusing on colorectal CRC patients who underwent radical resection. Despite this, the model’s efficacy was externally validated across an independent cohort showcasing diverse demographic and clinical features. Additionally, the assessment of LN by various pathologists introduces heterogeneity, potentially biasing the retrospective analysis outcomes. Lastly, MIF technique employed was relatively basic. Future studies incorporating more sophisticated methodologies, like single-cell RNA sequencing, are warranted to enrich our comprehension of the regional LNs’ significance.

Despite these limitations, our model still has many clinical advantages. First, the means to determine our ELNs-related subtypes are readily and widely available in current clinical settings, making this classification an easy-to-use tool that can be applied for individualized survival predictions. Second, this classification is a useful tool to evaluate the antitumor immune status of stage III CRC patients, also providing an important clinical basis for future research on the mechanism of antitumor immunity in LNs. Third, the model was validated in independent external validation cohorts, further allowing its more broad and routine application in various clinical settings.

## Conclusions

Despite advances in understanding the immune system’s role in tumor progression, the full extent of how regional lymph nodes contribute to systemic antitumor immunity remains elusive. To better address this knowledge gap, comprehensive learning of the series of cellular, molecular, and structural changes that occur within regional LNs may allow us to understand the complex mechanisms of host antitumor immunity. Until molecular assessment of the biological behavior of CRC becomes feasible, the model presented in this study could be a useful tool for evaluating the antitumor immune status and predicting the survival of stage III CRC patients in the clinical setting.

### Electronic supplementary material

Below is the link to the electronic supplementary material.

Supplementary Material 1

## Data Availability

The public datasets used in the present study could be accessed from SEER and TCGA database. Moreover, the data of FAH-SYSU database will be available upon reasonable request.

## Abbreviations

CRC: Colorectal cancer
ELNs: Examined lymph nodes
SEER: The surveillance, Epidemiology, and End results
RCS: Restricted cubic spline
FAH-SYSU: The first affiliated hospital, Sun yat-sen university
TCGA: The cancer genome atlas
LNs: Lymph nodes
OS: Overall survival
CSS: Cancer-specific survival
PFS: Progression-free survival
MIF: Multiplex immunofluorescence
AC: Adjuvant chemotherapy
AJCC: American joint committee on cancer
DEGs: Differentially expressed genes
IHC: Immunohistochemical
GO: Gene ontology
KEGG: Kyoto encyclopedia of genes and genomes
PSM: Propensity score matching
CI: Confidence interval
HR: Hazard ratio
Yes-AC: Receiving postoperative adjuvant chemotherapy
No-AC: Not receiving postoperative adjuvant chemotherapy
