# Supplementary material for: Prognostic and predictive value of examined lymph node count in stage III colorectal cancer: a population based study
Source: World J Surg Oncol. 2024 Jun 13;22:155. doi: 10.1186/s12957-024-03404-7 (PMC11170906; doi:10.1186/s12957-024-03404-7)
Supplement: Supplementary file 1 — Supplementary Material 1 [file 12957_2024_3404_MOESM1_ESM.docx]

**This supplementary material includes:**

**1. Supplementary Tables**

**Supplementary Table 1.** Univariable and multivariable Cox regression analysis in stage III CRC patients based on OS and CSS in train cohort.

| Characteristics | Overall survival | | Cancer-specific survival | |
| --- | --- | --- | --- | --- |
|  | univariable | multivariable | univariable | multivariable |
| Age at diagnosis (years old) |  |  |  |  |
| 20-49 | Ref | Ref | Ref | Ref |
| 50-69 | 1.39 (1.33-1.46, p<.001) | 1.36 (1.30-1.43, p<.001) | 1.19 (1.13-1.25, p<.001) | 1.19 (1.13-1.25, p<.001) |
| 70+ | 3.39 (3.24-3.55, p<.001) | 2.67 (2.54-2.80, p<.001) | 2.23 (2.12-2.34, p<.001) | 1.85 (1.76-1.95, p<.001) |
| Gender |  |  |  |  |
| Female | Ref | Ref | Ref | Ref |
| Male | 1.05 (1.02-1.07, p<.001) | 1.17 (1.15-1.20, p<.001) | 1.03 (1.00-1.06, p=.040) | 1.12 (1.09-1.15, p<.001) |
| Tumor location |  |  |  |  |
| Right colon | Ref | Ref | Ref | Ref |
| Left colon | 0.71 (0.69-0.73, p<.001) | 0.88 (0.86-0.91, p<.001) | 0.73 (0.71-0.76, p<.001) | 0.88 (0.85-0.91, p<.001) |
| Rectum | 0.75 (0.73-0.78, p<.001) | 0.96 (0.92-0.99, p=.022) | 0.85 (0.82-0.88, p<.001) | 0.99 (0.94-1.03, p=.613) |
| Tumor grade |  |  |  |  |
| Well/moderately | Ref | Ref | Ref | Ref |
| Poorly/undifferentiated | 1.52 (1.48-1.56, p<.001) | 1.30 (1.27-1.33, p<.001) | 1.66 (1.61-1.71, p<.001) | 1.39 (1.35-1.44, p<.001) |
| Histology type |  |  |  |  |
| Adenocarcinoma | Ref | Ref | Ref | Ref |
| Others | 1.07 (1.04-1.10, p<.001) | 1.08 (1.05-1.11, p<.001) | 1.08 (1.04-1.11, p<.001) | 1.10 (1.07-1.14, p<.001) |
| Tumor size (cm) |  |  |  |  |
| <3 | Ref | Ref | Ref | Ref |
| <=3&<5 | 1.29 (1.25-1.34, p<.001) | 1.11 (1.07-1.15, p<.001) | 1.38 (1.32-1.44, p<.001) | 1.12 (1.07-1.17, p<.001) |
| >=5 | 1.51 (1.46-1.56, p<.001) | 1.24 (1.19-1.28, p<.001) | 1.65 (1.58-1.72, p<.001) | 1.26 (1.21-1.32, p<.001) |
| Pathological T stage |  |  |  |  |
| T1/2 | Ref | Ref | Ref | Ref |
| T3/4 | 1.89 (1.82-1.97, p<.001) | 1.64 (1.57-1.71, p<.001) | 2.59 (2.45-2.74, p<.001) | 2.16 (2.04-2.29, p<.001) |
| Pathological N stage |  |  |  |  |
| N1 | Ref | Ref | Ref | Ref |
| N2 | 1.52 (1.48-1.55, p<.001) | 1.60 (1.57-1.64, p<.001) | 1.91 (1.85-1.96, p<.001) | 1.91 (1.86-1.97, p<.001) |
| Adjuvant radiotherapy |  |  |  |  |
| No | Ref | Ref | Ref | Ref |
| Yes | 0.86 (0.84-0.88, p<.001) | 1.09 (1.06-1.12, p<.001) | 0.96 (0.93-0.99, p=.005) | 1.12 (1.08-1.16, p<.001) |
| Adjuvant chemotherapy |  |  |  |  |
| No/unknown | Ref | Ref | Ref | Ref |
| Yes | 0.40 (0.39-0.40, p<.001) | 0.48 (0.47-0.49, p<.001) | 0.49 (0.47-0.50, p<.001) | 0.54 (0.52-0.56, p<.001) |
| ELNs count | 0.98 (0.98-0.98, p<.001) | 0.98 (0.98-0.98, p<.001) | 0.98 (0.98-0.98, p<.001) | 0.98 (0.98-0.98, p<.001) |

**NOTE.** Cox regression analyses are used to calculate the hazard ratio (HR) and 95% confidence interval (CI) based on OS and CSS. Covariables that are significant in univariable Cox regression analysis (P < 0.05) are included in the multivariable analysis.

**Supplementary Table 2.** Univariable and multivariable Cox regression analysis in stage III CRC patients based on OS and CSS in test cohort.

| Characteristics | Overall survival | | Cancer-specific survival | |
| --- | --- | --- | --- | --- |
|  | univariable | multivariable | univariable | multivariable |
| Age at diagnosis (years old) |  |  |  |  |
| 20-49 | Ref | Ref | Ref | Ref |
| 50-69 | 1.30 (1.23-1.38, p<.001) | 1.25 (1.18-1.33, p<.001) | 1.09 (1.03-1.16, p=.005) | 1.08 (1.01-1.15, p=.015) |
| 70+ | 3.19 (3.02-3.37, p<.001) | 2.46 (2.32-2.60, p<.001) | 2.08 (1.96-2.21, p<.001) | 1.70 (1.59-1.81, p<.001) |
| Gender |  |  |  |  |
| Female | Ref | Ref | Ref | Ref |
| Male | 1.05 (1.02-1.08, p<.001) | 1.16 (1.12-1.19, p<.001) | 1.02 (0.99-1.06, p=.167) |  |
| Tumor location |  |  |  |  |
| Right colon | Ref | Ref | Ref | Ref |
| Left colon |  | 0.89 (0.86-0.93, p<.001) | 0.75 (0.72-0.78, p<.001) | 0.89 (0.85-0.93, p<.001) |
| Rectum |  | 0.98 (0.94-1.03, p=.452) | 0.84 (0.80-0.88, p<.001) | 1.07 (1.01-1.12, p=.012) |
| Tumor grade |  |  |  |  |
| Well/moderately | Ref | Ref | Ref | Ref |
| Poorly/undifferentiated | 1.50 (1.46-1.55, p<.001) | 1.28 (1.24-1.32, p<.001) | 1.64 (1.58-1.70, p<.001) | 1.36 (1.31-1.41, p<.001) |
| Histology type |  |  |  |  |
| Adenocarcinoma | Ref | Ref | Ref | Ref |
| Others | 1.06 (1.03-1.10, p<.001) | 1.06 (1.02-1.09, p=.001) | 1.08 (1.04-1.12, p<.001) | 1.08 (1.04-1.13, p<.001) |
| Tumor size (cm) |  |  |  |  |
| <3 | Ref | Ref | Ref | Ref |
| <=3&<5 | 1.32 (1.26-1.37, p<.001) | 1.13 (1.08-1.18, p<.001) | 1.47 (1.39-1.55, p<.001) | 1.18 (1.12-1.25, p<.001) |
| >=5 | 1.55 (1.49-1.62, p<.001) | 1.27 (1.21-1.32, p<.001) | 1.78 (1.69-1.88, p<.001) | 1.33 (1.26-1.41, p<.001) |
| Pathological T stage |  |  |  |  |
| T1/2 | Ref | Ref | Ref | Ref |
| T3/4 | 1.91 (1.82-2.01, p<.001) | 1.64 (1.56-1.73, p<.001) | 2.73 (2.54-2.92, p<.001) | 2.23 (2.07-2.40, p<.001) |
| Pathological N stage |  |  |  |  |
| N1 | Ref | Ref | Ref | Ref |
| N2 | 1.52 (1.48-1.56, p<.001) | 1.58 (1.54-1.63, p<.001) | 1.91 (1.85-1.98, p<.001) | 1.89 (1.82-1.96, p<.001) |
| Adjuvant radiotherapy |  |  |  |  |
| No | Ref | Ref | Ref | Ref |
| Yes | 0.87 (0.85-0.90, p<.001) | 1.08 (1.04-1.12, p<.001) | 0.99 (0.95-1.02, p=.409) |  |
| Adjuvant chemotherapy |  |  |  |  |
| No/unknown | Ref | Ref | Ref | Ref |
| Yes | 0.40 (0.39-0.41, p<.001) | 0.49 (0.47-0.50, p<.001) | 0.50 (0.49-0.52, p<.001) | 0.57 (0.55-0.59, p<.001) |
| ELNs count | 0.98 (0.98-0.98, p<.001) | 0.98 (0.98-0.98, p<.001) | 0.98 (0.98-0.99, p<.001) | 0.98 (0.97-0.98, p<.001) |

**NOTE.** Cox regression analyses are used to calculate the hazard ratio (HR) and 95% confidence interval (CI) based on OS and CSS. Covariables that are significant in univariable Cox regression analysis (P < 0.05) are included in the multivariable analysis.

**Supplementary Table 3.** Univariable and multivariable Cox regression analysis in stage III CRC patients based on OS in FAH-SYSU cohort.

| Characteristics | Overall survival | |
| --- | --- | --- |
|  | HR (univariable) | HR (multivariable) |
| Age at diagnosis (years old) |  |  |
| 20-49 | Ref | Ref |
| 50-69 | 1.28 (1.06-1.55, p=.012) | 1.21 (0.97-1.50, p=.086) |
| 70+ | 2.04 (1.66-2.51, p<.001) | 1.75 (1.35-2.27, p<.001) |
| Gender |  |  |
| Female | Ref | Ref |
| Male | 0.82 (0.71-0.95, p=.007) | 0.88 (0.74-1.05, p=.165) |
| Tumor location |  |  |
| Right colon | Ref | Ref |
| Left colon | 0.85 (0.69-1.04, p=.113) | 0.87 (0.67-1.13, p=.295) |
| Rectum | 1.33 (1.11-1.60, p=.002) | 1.47 (1.16-1.85, p=.001) |
| Tumor grade |  |  |
| Well/moderately | Ref | Ref |
| Poorly/undifferentiated | 1.82 (1.53-2.16, p<.001) | 1.74 (1.39-2.19, p<.001) |
| Histology type |  |  |
| Adenocarcinoma | Ref | Ref |
| Others | 1.24 (0.94-1.65, p=.131) |  |
| Tumor size (cm) |  |  |
| <3 | Ref | Ref |
| <=3&<5 | 1.13 (0.87-1.45, p=.356) | 0.82 (0.61-1.09, p=.167) |
| >=5 | 1.29 (1.00-1.66, p=.046) | 1.09 (0.81-1.46, p=.579) |
| Pathological T stage |  |  |
| T1/2 | Ref | Ref |
| T3/4 | 2.12 (1.56-2.90, p<.001) | 1.86 (1.28-2.72, p=.001) |
| Pathological N stage |  |  |
| N1 | Ref | Ref |
| N2 | 1.83 (1.58-2.11, p<.001) | 1.88 (1.56-2.26, p<.001) |
| Adjuvant chemotherapy |  |  |
| No/unknown | Ref | Ref |
| Yes | 0.66 (0.54-0.79, p<.001) | 0.69 (0.56-0.85, p=.001) |
| ELNs count | 0.82 (0.75-0.89, p<.001) | 0.81 (0.72-0.91, p<.001) |

**NOTE.** Cox regression analyses are used to calculate the hazard ratio (HR) and 95% confidence interval (CI) based on OS. Covariables that are significant in univariable Cox regression analysis (P < 0.05) are included in the multivariable analysis.

**Supplementary Table 4.** Subgroup analysis of ELNs in stage III CRC patients based on OS in train and test cohorts.

| Subgroups | HR (95%CI, P-value) for OS |
| --- | --- |
| Train cohort |  |
| Pathological N1 stage | 0.98 (0.98-0.98, p<.001) |
| Pathological N2 stage | 0.99 (0.98-0.99, p<.001) |
|  |  |
| Test cohort |  |
| Pathological N1 stage | 0.98 (0.98-0.98, p<.001) |
| Pathological N2 stage | 0.99 (0.98-0.99, p<.001) |

**NOTE.** Cox regression analyses are used to calculate the hazard ratio (HR) and 95% confidence interval (CI) based on OS.

**Supplementary Table 5.** Univariable and multivariable Cox regression analysis in stage III CRC patients based on OS in train cohort.

| Characteristics | Train cohort | |
| --- | --- | --- |
|  | univariable | multivariable |
| Age at diagnosis (years old) |  |  |
| 20-49 | Ref | Ref |
| 50-69 | 1.39 (1.33-1.46, p<.001) | 1.38 (1.31-1.44, p<.001) |
| 70+ | 3.39 (3.24-3.55, p<.001) | 2.70 (2.57-2.83, p<.001) |
| Gender |  |  |
| Female | Ref | Ref |
| Male | 1.05 (1.02-1.07, p<.001) | 1.18 (1.15-1.20, p<.001) |
| Tumor location |  |  |
| Right colon | Ref | Ref |
| Left colon | 0.71 (0.69-0.73, p<.001) | 0.88 (0.85-0.90, p<.001) |
| Rectum | 0.75 (0.73-0.78, p<.001) | 1.00 (0.97-1.03, p=.954) |
| Tumor grade |  |  |
| Well/moderately | Ref | Ref |
| Poorly/undifferentiated | 1.52 (1.48-1.56, p<.001) | 1.30 (1.27-1.33, p<.001) |
| Histology type |  |  |
| Adenocarcinoma | Ref | Ref |
| Others | 1.07 (1.04-1.10, p<.001) | 1.08 (1.05-1.11, p<.001) |
| Tumor size (cm) |  |  |
| <3 | Ref | Ref |
| <=3&<5 | 1.29 (1.25-1.34, p<.001) | 1.11 (1.07-1.15, p<.001) |
| >=5 | 1.51 (1.46-1.56, p<.001) | 1.23 (1.19-1.28, p<.001) |
| Pathological T stage |  |  |
| T1/2 | Ref | Ref |
| T3/4 | 1.89 (1.82-1.97, p<.001) | 1.65 (1.58-1.72, p<.001) |
| Pathological N stage |  |  |
| N1 | Ref | Ref |
| N2 | 1.52 (1.48-1.55, p<.001) | 1.60 (1.56-1.64, p<.001) |
| Adjuvant chemotherapy |  |  |
| No/unknown | Ref | Ref |
| Yes | 0.40 (0.39-0.40, p<.001) | 0.49 (0.47-0.50, p<.001) |
| ELNs related subtypes |  |  |
| SN-ELN | Ref | Ref |
| MN-ELN | 0.83 (0.80-0.86, p<.001) | 0.80 (0.77-0.82, p<.001) |
| LN-ELN | 0.70 (0.68-0.72, p<.001) | 0.67 (0.65-0.69, p<.001) |

**NOTE.** Cox regression analyses are used to calculate the hazard ratio (HR) and 95% confidence interval (CI) based on OS. Covariables that are significant in univariable Cox regression analysis (P < 0.05) are included in the multivariable analysis.

**Supplementary Table 6.** Univariable and multivariable Cox regression analysis in stage III CRC patients based on OS in test cohort.

| Characteristics | Test cohort | |
| --- | --- | --- |
|  | univariable | multivariable |
| Age at diagnosis (years old) |  |  |
| 20-49 | Ref | Ref |
| 50-69 | 1.30 (1.23-1.38, p<.001) | 1.27 (1.20-1.35, p<.001) |
| 70+ | 3.19 (3.02-3.37, p<.001) | 2.50 (2.36-2.65, p<.001) |
| Gender |  |  |
| Female | Ref | Ref |
| Male | 1.05 (1.02-1.08, p<.001) | 1.16 (1.12-1.19, p<.001) |
| Tumor location |  |  |
| Right colon | Ref | Ref |
| Left colon | 0.73 (0.71-0.75, p<.001) | 0.89 (0.86-0.92, p<.001) |
| Rectum | 0.76 (0.73-0.79, p<.001) | 1.03 (0.98-1.07, p=.224) |
| Tumor grade |  |  |
| Well/moderately | Ref | Ref |
| Poorly/undifferentiated | 1.50 (1.46-1.55, p<.001) | 1.28 (1.24-1.32, p<.001) |
| Histology type |  |  |
| Adenocarcinoma | Ref | Ref |
| Others | 1.06 (1.03-1.10, p<.001) | 1.05 (1.02-1.09, p=.002) |
| Tumor size (cm) |  |  |
| <3 | Ref | Ref |
| <=3&<5 | 1.32 (1.26-1.37, p<.001) | 1.13 (1.08-1.18, p<.001) |
| >=5 | 1.55 (1.49-1.62, p<.001) | 1.26 (1.21-1.32, p<.001) |
| Pathological T stage |  |  |
| T1/2 | Ref | Ref |
| T3/4 | 1.91 (1.82-2.01, p<.001) | 1.64 (1.56-1.73, p<.001) |
| Pathological N stage |  |  |
| N1 | Ref | Ref |
| N2 | 1.52 (1.48-1.56, p<.001) | 1.58 (1.54-1.63, p<.001) |
| Adjuvant chemotherapy |  |  |
| No/unknown | Ref | Ref |
| Yes | 0.40 (0.39-0.41, p<.001) | 0.49 (0.48-0.51, p<.001) |
| ELNs related subtypes |  |  |
| SN-ELN | Ref | Ref |
| MN-ELN | 0.81 (0.78-0.84, p<.001) | 0.79 (0.76-0.82, p<.001) |
| LN-ELN | 0.68 (0.66-0.70, p<.001) | 0.64 (0.61-0.66, p<.001) |

**NOTE.** Cox regression analyses are used to calculate the hazard ratio (HR) and 95% confidence interval (CI) based on OS. Covariables that are significant in univariable Cox regression analysis (P < 0.05) are included in the multivariable analysis.

**Supplementary Table 7.** Univariable and multivariable Cox regression analysis in stage III CRC patients based on OS in FAH-SYSU cohort.

| Characteristics | FAH-SYSU cohort | |
| --- | --- | --- |
|  | univariable | multivariable |
| Age at diagnosis (years old) |  |  |
| 20-49 | Ref | Ref |
| 50-69 | 1.28 (1.06-1.55, p=.012) | 1.21 (0.98-1.51, p=.082) |
| 70+ | 2.04 (1.66-2.51, p<.001) | 1.76 (1.36-2.28, p<.001) |
| Gender |  |  |
| Female | Ref | Ref |
| Male | 0.82 (0.71-0.95, p=.007) | 0.88 (0.74-1.05, p=.155) |
| Tumor location |  |  |
| Right colon | Ref | Ref |
| Left colon | 0.85 (0.69-1.04, p=.113) | 0.87 (0.67-1.14, p=.308) |
| Rectum | 1.33 (1.11-1.60, p=.002) | 1.48 (1.17-1.86, p=.001) |
| Tumor grade |  |  |
| Well/moderately | Ref | Ref |
| Poorly/undifferentiated | 1.82 (1.53-2.16, p<.001) | 1.73 (1.38-2.18, p<.001) |
| Histology type |  |  |
| Adenocarcinoma | Ref | Ref |
| Others | 1.24 (0.94-1.65, p=.131) |  |
| Tumor size (cm) |  |  |
| <3 | Ref | Ref |
| <=3&<5 | 1.13 (0.87-1.45, p=.356) | 0.82 (0.61-1.09, p=.168) |
| >=5 | 1.29 (1.00-1.66, p=.046) | 1.09 (0.81-1.46, p=.574) |
| Pathological T stage |  |  |
| T1/2 | Ref | Ref |
| T3/4 | 2.12 (1.56-2.90, p<.001) | 1.87 (1.28-2.72, p=.001) |
| Pathological N stage |  |  |
| N1 | Ref | Ref |
| N2 | 1.83 (1.58-2.11, p<.001) | 1.89 (1.57-2.27, p<.001) |
| Adjuvant chemotherapy |  |  |
| No/unknown | Ref | Ref |
| Yes | 0.66 (0.54-0.79, p<.001) | 0.69 (0.56-0.86, p=.001) |
| ELNs related subtypes |  |  |
| SN-ELN | Ref | Ref |
| MN-ELN | 0.83 (0.68-1.01, p=.063) | 0.74 (0.58-0.94, p=.014) |
| LN-ELN | 0.67 (0.56-0.79, p<.001) | 0.65 (0.52-0.81, p<.001) |

**NOTE.** Cox regression analyses are used to calculate the hazard ratio (HR) and 95% confidence interval (CI) based on OS. Covariables that are significant in univariable Cox regression analysis (P < 0.05) are included in the multivariable analysis.

**Supplementary Table 8.** Univariable and multivariable Cox regression analysis in stage III CRC patients based on OS in TCGA cohort.

| Characteristics | TCGA cohort | |
| --- | --- | --- |
|  | univariable | multivariable |
| Age at diagnosis (years old) |  |  |
| 20-49 | Ref | Ref |
| 50-69 | 2.86 (0.64-12.75, p=.168) | 2.36 (0.50-11.06, p=.277) |
| 70+ | 5.76 (1.33-24.97, p=.019) | 4.67 (1.04-21.02, p=.045) |
| Gender |  |  |
| Female | Ref | Ref |
| Male | 0.61 (0.30-1.24, p=.174) | 0.43 (0.21-0.90, p=.025) |
| Tumor location |  |  |
| Right colon | Ref | Ref |
| Left colon | 0.89 (0.39-2.04, p=.780) |  |
| Rectum | 1.09 (0.49-2.43, p=.833) |  |
| Histology type |  |  |
| Adenocarcinoma | Ref | Ref |
| Others | 0.93 (0.33-2.65, p=.891) |  |
| Pathological T stage |  |  |
| T1/2 | Ref | Ref |
| T3/4 | 1.28 (0.31-5.39, p=.733) |  |
| Pathological N stage |  |  |
| N1 | Ref | Ref |
| N2 | 2.66 (1.34-5.27, p=.005) | 3.23 (1.53-6.80, p=.002) |
| ELNs related subtypes |  |  |
| SN-ELN | Ref | Ref |
| MN-ELN | 0.79 (0.32-1.97, p=.611) | 0.58 (0.22-1.50, p=.260) |
| LN-ELN | 0.26 (0.10-0.67, p=.005) | 0.20 (0.08-0.55, p=.002) |

**NOTE.** Cox regression analyses are used to calculate the hazard ratio (HR) and 95% confidence interval (CI) based on OS. Covariables that are significant in univariable Cox regression analysis (P < 0.05) are included in the multivariable analysis.

**Supplementary Table 9.** Comparisons of baseline characteristics in patients with stage III CRC between postoperative adjuvant chemotherapy (AC) and No-AC group within LN-ELN group from FAH-SYSU cohort before PSM and after PSM respectively.

| Characteristics | Before PSM | |  | After PSM | |  |
| --- | --- | --- | --- | --- | --- | --- |
|  | No-AC  n=128 | Yes-AC  n=991 | p-value | No-AC  n=107 | Yes-AC  n=107 | p-value |
| Age at diagnosis, n (%), years old |  |  |  |  |  |  |
| 20-49 | 36 (28.12) | 264 (26.64) | <0.001 | 28 (26.17) | 28 (26.17) | 1.000 |
| 50-69 | 55 (42.97) | 620 (62.56) |  | 50 (46.73) | 50 (46.73) |  |
| 70+ | 37 (28.91) | 107 (10.80) |  | 29 (27.10) | 29 (27.10) |  |
| Gender, n (%) |  |  |  |  |  |  |
| Female | 73 (57.03) | 534 (53.88) | 0.563 | 63 (58.88) | 63 (58.88) | 1.000 |
| Male | 55 (42.97) | 457 (46.12) |  | 44 (41.12) | 44 (41.12) |  |
| Tumor location, n (%) |  |  |  |  |  |  |
| Right colon | 46 (35.94) | 292 (29.47) | 0.029 | 39 (36.45) | 39 (36.45) | 1.000 |
| Left colon | 29 (22.66) | 340 (34.31) |  | 26 (24.30) | 26 (24.30) |  |
| Rectum | 53 (41.41) | 359 (36.23) |  | 42 (39.25) | 42 (39.25) |  |
| Tumor grade, n (%) |  |  |  |  |  |  |
| Well/moderately | 119 (92.97) | 665 (78.14) | <0.001 | 103 (96.26) | 103 (96.26) | 1.000 |
| Poorly/undifferentiated | 9 (7.03) | 186 (21.86) |  | 4 (3.74) | 4 (3.74) |  |
| Histology type, n (%) |  |  |  |  |  |  |
| Adenocarcinoma | 126 (98.44) | 781 (91.67) | 0.011 | 107 (100.00) | 107 (100.00) | 1.000 |
| Others | 2 (1.56) | 71 (8.33) |  | 0 (0) | 0 (0) |  |
| Tumor size, n (%), cm |  |  |  |  |  |  |
| <3 | 13 (10.32) | 98 (9.99) | 0.987 | 9 (8.41) | 9 (8.41) | 1.000 |
| <=3&<5 | 48 (38.10) | 380 (38.74) |  | 38 (35.51) | 38 (35.51) |  |
| >=5 | 65 (51.59) | 503 (51.27) |  | 60 (56.07) | 60 (56.07) |  |
| Pathological T stage, n (%) |  |  |  |  |  | 1.000 |
| T1/2 | 15 (11.72) | 72 (7.27) | 0.111 | 5 (4.67) | 5 (4.67) |  |
| T3/4 | 113 (88.28) | 919 (92.73) |  | 102 (95.33) | 102 (95.33) |  |
| Pathological N stage, n (%) |  |  |  |  |  | 1.000 |
| N1 | 80 (62.50) | 651 (65.69) | 0.538 | 69 (64.49) | 69 (64.49) |  |
| N2 | 48 (37.50) | 340 (34.31) |  | 38 (35.51) | 38 (35.51) |  |

**NOTE.** P values were calculated using the χ^2^ test for categorical variables.

**Supplementary Table 10.** Comparisons of baseline characteristics in patients with stage III CRC between postoperative adjuvant chemotherapy (AC) and No-AC group within MN-ELN group from FAH-SYSU cohort before PSM and after PSM respectively.

| Characteristics | Before PSM | |  | After PSM | |  |
| --- | --- | --- | --- | --- | --- | --- |
|  | No-AC  n=70 | Yes-AC  n=374 |  | No-AC  n=58 | Yes-AC  n=58 |  |
| Age at diagnosis, n (%), years old |  |  |  |  |  |  |
| 20-49 | 12 (17.14) | 84 (22.46) | 0.001 | 10 (17.24) | 6 (10.34) | 0.526 |
| 50-69 | 30 (42.86) | 219 (58.56) |  | 26 (44.83) | 30 (51.72) |  |
| 70+ | 28 (40.00) | 71 (18.98) |  | 22 (37.93) | 22 (37.93) |  |
| Gender, n (%) |  |  |  |  |  |  |
| Female | 42 (60.00) | 238 (63.64) | 0.657 | 35 (60.34) | 35 (60.34) | 1 |
| Male | 28 (40.00) | 136 (36.36) |  | 23 (39.66) | 23 (39.66) |  |
| Tumor location, n (%) |  |  |  |  |  |  |
| Right colon | 15 (21.43) | 41 (10.96) | <0.001 | 12 (20.69) | 11 (18.97) | 0.657 |
| Left colon | 10 (14.29) | 151 (40.37) |  | 10 (17.24) | 14 (24.14) |  |
| Rectum | 45 (64.29) | 182 (48.66) |  | 36 (62.07) | 33 (56.90) |  |
| Tumor grade, n (%) |  |  |  |  |  |  |
| Well/moderately | 67 (95.71) | 274 (82.04) | 0.007 | 57 (98.28) | 56 (96.55) | 1 |
| Poorly/undifferentiated | 3 (4.29) | 60 (17.96) |  | 1 (1.72) | 2 (3.45) |  |
| Histology type, n (%) |  |  |  |  |  |  |
| Adenocarcinoma | 70 (100.00) | 307 (91.92) | 0.028 | 58 (100.00) | 58 (100.00) | 1 |
| Others | 0 (0.00) | 27 (8.08) |  | 0 (0) | 0 (0) |  |
| Tumor size, n (%), cm |  |  |  |  |  |  |
| <3 | 7 (10.00) | 55 (14.78) | 0.432 | 6 (10.34) | 5 (8.62) | 0.756 |
| <=3&<5 | 33 (47.14) | 182 (48.92) |  | 27 (46.55) | 31 (53.45) |  |
| >=5 | 30 (42.86) | 135 (36.29) |  | 25 (43.10) | 22 (37.93) |  |
| Pathological T stage, n (%) |  |  |  |  |  |  |
| T1/2 | 6 (8.57) | 46 (12.30) | 0.492 | 3 (5.17) | 4 (6.90) | 1 |
| T3/4 | 64 (91.43) | 328 (87.70) |  | 55 (94.83) | 54 (93.10) |  |
| Pathological N stage, n (%) |  |  |  |  |  |  |
| N1 | 56 (80.00) | 256 (68.45) | 0.072 | 51 (87.93) | 52 (89.66) | 1 |
| N2 | 14 (20.00) | 118 (31.55) |  | 7 (12.07) | 6 (10.34) |  |

**NOTE.** P values were calculated using the χ^2^ test for categorical variables.

**Supplementary Table 11.** Comparisons of baseline characteristics in patients with stage III CRC between postoperative adjuvant chemotherapy (AC) and No-AC group within SN-ELN group from FAH-SYSU cohort before PSM and after PSM respectively.

| Characteristics | Before PSM | |  | After PSM | |  |
| --- | --- | --- | --- | --- | --- | --- |
|  | No-AC  n=122 | Yes-AC  n=278 |  | No-AC  n=76 | Yes-AC  n=76 |  |
| Age at diagnosis, n (%), years old |  |  |  |  |  |  |
| 20-49 | 14 (11.48) | 67 (24.10) | <0.001 | 11 (14.47) | 13 (17.11) | 0.905 |
| 50-69 | 51 (41.80) | 166 (59.71) |  | 41 (53.95) | 40 (52.63) |  |
| 70+ | 57 (46.72) | 45 (16.19) |  | 24 (31.58) | 23 (30.26) |  |
| Gender, n (%) |  |  |  |  |  |  |
| Female | 68 (55.74) | 174 (62.59) | 0.238 | 46 (60.53) | 42 (55.26) | 0.622 |
| Male | 54 (44.26) | 104 (37.41) |  | 30 (39.47) | 34 (44.74) |  |
| Tumor location, n (%) |  |  |  |  |  |  |
| Right colon | 22 (18.03) | 24 (8.63) | 0.022 | 9 (11.84) | 9 (11.84) | 0.758 |
| Left colon | 40 (32.79) | 110 (39.57) |  | 23 (30.26) | 19 (25.00) |  |
| Rectum | 60 (49.18) | 144 (51.80) |  | 44 (57.89) | 48 (63.16) |  |
| Tumor grade, n (%) |  |  |  |  |  |  |
| Well/moderately | 117 (95.90) | 212 (81.54) | <0.001 | 75 (98.68) | 75 (98.68) | 1 |
| Poorly/undifferentiated | 5 (4.10) | 48 (18.46) |  | 1 (1.32) | 1 (1.32) |  |
| Histology type, n (%) |  |  |  |  |  |  |
| Adenocarcinoma | 120 (98.36) | 249 (95.77) | 0.317 | 76 (100.00) | 76 (100.00) | 1 |
| Others | 2 (1.64) | 11 (4.23) |  | 0 (0) | 0 (0) |  |
| Tumor size, n (%), cm |  |  |  |  |  |  |
| <3 | 15 (12.61) | 57 (21.03) | 0.135 | 13 (17.11) | 10 (13.16) | 0.733 |
| <=3&<5 | 68 (57.14) | 136 (50.18) |  | 45 (59.21) | 45 (59.21) |  |
| >=5 | 36 (30.25) | 78 (28.78) |  | 18 (23.68) | 21 (27.63) |  |
| Pathological T stage, n (%) |  |  |  |  |  |  |
| T1/2 | 16 (13.11) | 42 (15.11) | 0.714 | 8 (10.53) | 12 (15.79) | 0.472 |
| T3/4 | 106 (86.89) | 236 (84.89) |  | 68 (89.47) | 64 (84.21) |  |
| Pathological N stage, n (%) |  |  |  |  |  |  |
| N1 | 107 (87.70) | 229 (82.37) | 0.234 | 68 (89.47) | 71 (93.42) | 0.562 |
| N2 | 15 (12.30) | 49 (17.63) |  | 8 (10.53) | 5 (6.58) |  |

**NOTE.** P values were calculated using the χ^2^ test for categorical variables.

**2. Supplementary Figures**


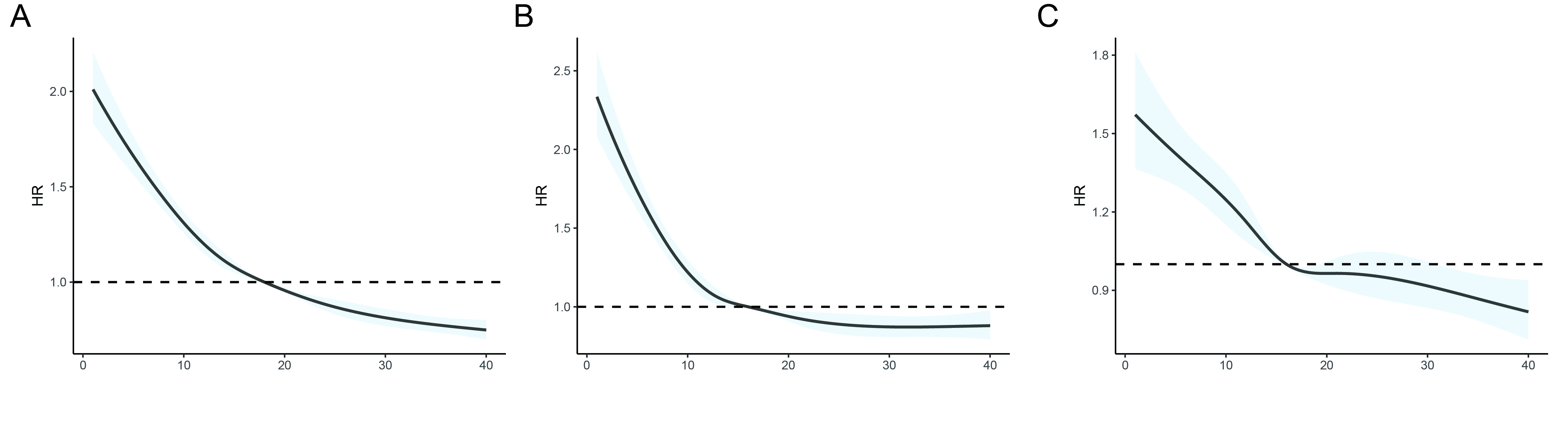


**Supplementary Figure1.** Cubic spline graph of the adjusted HR (represented by solid line) and 95% CI (represented by the blue area) for the association between ELNs count and OS. (A) Log hazard ratio function and 95 % pointwise confidence band estimated by a restricted cubic spline function for quantifying the effect of ELNs counts on right colon cancer. Smaller log hazard ratios indicate better survival. (B) Log hazard ratio function and 95 % pointwise confidence band estimated by a restricted cubic spline function for quantifying the effect of ELNs counts on left colon cancer. (C) Log hazard ratio function and 95 % pointwise confidence band estimated by a restricted cubic spline function for quantifying the effect of ELNs counts on rectal cancer.


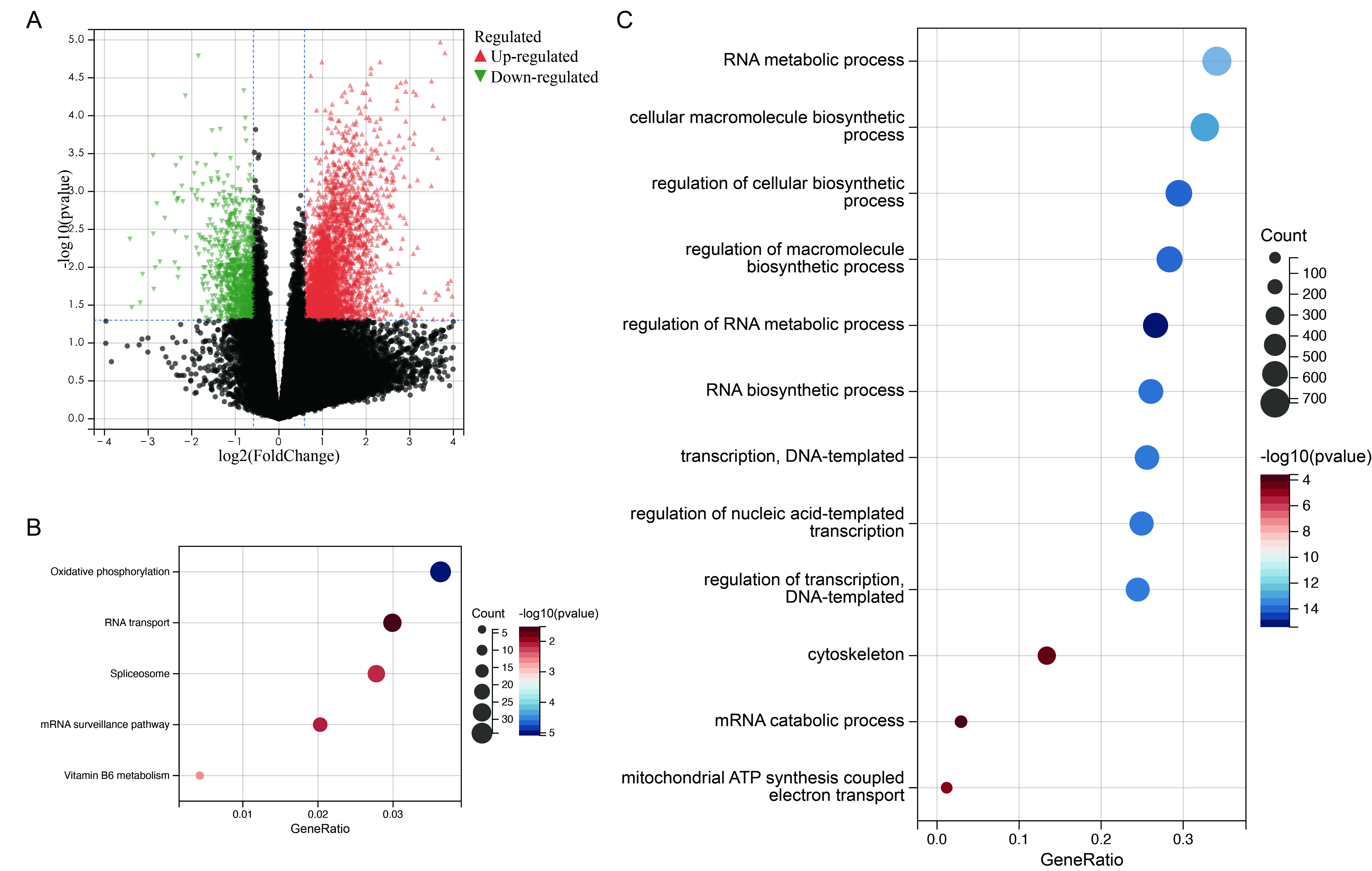


**Supplementary Figure2.** The analysis on potential biological mechanisms among different ELNs-related subtypes. (A) The volcano plot showed the differentially expressed genes (DEGs) between SN-ELN and MN-ELN groups. Red points: up-expression DEGs in MN-ELN with log2-fold change > 0.5 and P < 0.05; Green points: down-expression DEGs in MN-ELN with log2-fold change < -0.5 and P < 0.05; Black point: gene expression with |log2-fold change| < 0.5 or P < 0.05. (B-C) The GO and KEGG analyzed presented the enrichment biological pathways between SN-ELN and MN-ELN groups.
